# Neonatal stunting and early childhood caries: A mini-review

**DOI:** 10.3389/fped.2022.871862

**Published:** 2022-07-18

**Authors:** Arlette Suzy Setiawan, Ratna Indriyanti, Netty Suryanti, Laili Rahayuwati, Neti Juniarti

**Affiliations:** ^1^Department of Pediatric Dentistry, Faculty of Dentistry, Universitas Padjadjaran, Sumedang, Indonesia; ^2^Department of Community Dentistry, Faculty of Dentistry, Universitas Padjadjaran, Sumedang, Indonesia; ^3^Department of Community Nursing, Faculty of Nursing, Universitas Padjadjaran, Sumedang, Indonesia

**Keywords:** stunting, neonatal, at birth, early childhood caries, low birth weight

## Abstract

The nutritional status of pregnant women greatly determines their newborn outcome. Deficiencies of several micronutrients are associated with stunting in early childhood, affecting health into adulthood. However, apart from the systemic disease that has been a concern so far, fetal undernutrition can also be associated with dental caries in a child's early life, especially since the primary teeth begin to form during the mother's gestation period. The important thing to underline regarding the intrauterine formation of primary teeth is especially in terms of enamel formation. One of the causes of developmental enamel defects that will contribute to the emergence of early childhood caries is the malnutrition of the pregnant mother. This mini-review aims to understand the linkage mechanism behind neonatal stunting to early childhood caries. This concept is expected to generate further research to help prevent both growth stunting and early childhood caries. In addition, with some evidence-based research, the importance of the first dental visit can be further promoted.

## Introduction

Early childhood malnutrition, including fetal stunting, stunting, wasting, vitamin A and zinc deficiencies, is a global problem with multiple impacts, affecting the survival of individuals and communities (1). High morbidity in some low- and middle-income countries can lead to a significant increase in mortality and thus a global burden of disease (2). Among the various forms of nutritional problems, wasting and stunting are more common and are the leading cause of malnutrition-related morbidity and mortality in children. Nearly 178 million children under five are affected by stunting globally, 90% of whom live in 36 low- and middle-income countries (1, 3). Stunting has severe long-term sequelae, including an increased risk of morbidity and mortality and retarded psychomotor development. The treatment of stunted children is still a challenge because the underlying etiology and pathophysiological mechanisms are still elusive (4).

Approaching the age of one century, Indonesia (5) turned out to be one of the 36 countries facing this severe nutritional problem. Data collected by WHO and released in 2018 on the prevalence of stunted children shows that Indonesia ranks in the top three in South Asia, after Timor-Leste (50.5%) and India (36.4%) (6). Stunting is a form of malnutrition characterized by height one standard deviation below the WHO median (z-score <-2 S.D.) (3, 7). This stunting phenomenon has received international attention for two main reasons; first, it affects many children worldwide, and second, it has serious short- and long-term health consequences (1). In addition, stunting has become the main WHO target to reduce the high burden of malnutrition-related diseases by reducing stunting by 40% globally by 2025 (3, 7). The Indonesian government is targeting a reduction to 14% by 2024 to produce a productive golden generation in 2045, on the 100th anniversary of its independence (8).

According to the UNICEF framework, three main factors contribute to growth retardation; unbalanced diet, low birth weight, and medical history (1). Relevant to this framework and considering the growth and development of the child from the intrauterine to adulthood, it is crucial to treat feeding delays from infancy, especially in the first 1,000 days of life. This golden growth period includes 270 days of pregnancy and 730 days after birth (9). Inadequate nutritional care during this period can lead to stunted growth in children, not only growth retardation (7).

Maternal physical factors are very influential on fetal growth. Maternal weight (before pregnancy), maternal height, and weight gain during pregnancy are directly related to maternal nutrition and fetal growth. Maternal nutrition significantly affects fetal growth, especially in developing countries. Although many factors interact and influence fetal development, maternal malnutrition is considered a significant cause of intrauterine growth retardation IUGR in developing countries (10).

In addition to pre-term birth, IUGR can also lead to low birth weight (LBW). Maternal infection and low food intake are strong predictors of this growth retardation (11, 12). Infection not only reduces appetite and food intake but also affects the nutrient intake of the mother, in addition to possibly causing increased metabolic stress, leading to higher nutritional requirements. On the other hand, IUGR is triggered when the mother's energy and protein intake are affected. Underweight mothers are 6.5 times more likely to have LBW babies than well-nourished mothers (13). Most fetal weight gain occurs in the third trimester, but the effects of these nutrients are not limited to the second or third trimester. Animal studies have shown that nutritional deficiencies surrounding the period of peri-implantation can significantly impair fetal growth (14). Infection and malnutrition complement each other in a vicious cycle of pregnancy, leading to adverse obstetric outcomes, including low birth weight, which affects the incidence of infant growth retardation. In addition, babies with LBW experience intrauterine growth restriction, resulting in slow growth and often unable to keep up with the growth rates that must be achieved after birth. Therefore, IUGR affects growth arrest, resulting in stunted growth. Insufficient catch-up growth also affects the incidence of stunting (15). Therefore, continuous care during the perinatal period, pregnancy and lactation can ensure the best outcomes and health of the newborn. Babies are expected to catch up in the first few months of life without further complications (16).

Intrauterine malnutrition can affect the growth and development of various organs in the child's body, including the growth and development of teeth. Although malnourished children often experience delayed tooth eruption, dental caries are very high in early childhood, especially when malnutrition occurs during tooth formation (17). Growth stunting children in Indonesia have experienced primary caries, and most of them are of high severity. Furthermore, caries and growth retardation in children's deciduous teeth are linked (18). Data show that in 2013, about 24% of babies in Indonesia were born with stunted growth, and in 2018, the proportion of children under the age of five suffering from dental caries was 90% (19, 20). In this mini-review, we provide a brief overview of the consequences of neonatal growth retardation due to fetal malnutrition during tooth development and its contribution to the development of early childhood caries.

## Neonatal stunting

Neonatal stunting, also known as stunting at birth, is a type of chronic malnutrition experienced by children, mainly related to prenatal malnutrition that persists and continues into the following years (21).

A shorter length characterizes neonatal stunting for gestational age, (22) is a severe syndrome of physical and cognitive impairment with irreversible and recurring sequelae since conception (23, 24). The short-term and long-term effects are not limited to this child's life but can pass to the next generation. Babies of malnourished mothers are four times more likely to be born stunted and twice as likely to be born at 3 months and 2 years old (22, 25, 26).

When teens become mothers who experience malnutrition and anemia, newborns are likely to be stunted before conception (27). Malnutrition during pregnancy and mothers living in unsanitary conditions are made worse. Forty-six-point six percent of adolescent girls aged 15–19 in Indonesia suffer from chronic energy deficiency disorder (CED). During pregnancy, 24.2% of women of childbearing age between the ages of 15 and 49 were at risk of CED, and 37.1% were at risk of anemia. Most pregnant women (urban and rural) have food intake, energy, and protein problems. These conditions accompany pregnant women, who are also typically short (<150 cm), in 31.3% of cases, resulting in babies with a birth weight of less than 2,500 g and a body length of <48 cm. If we pool together children with birth weight <2,500 g and body length <48 cm, the proportion in Indonesia is about 4.3% (Table 1) (28).

**Table 1:** Data on conditions that potentially as predictors of neonatal stunting in Indonesia (28).

**Predictors**	**Percentage in 2017**
Percentage of adolescent girls with short nutritional status	27.6%
Percentage of adolescent girls with concise nutritional status	7.9%
Percentage of adolescent girls with CED	32%
Percentage of pregnant women CED	14.8%
Presentation of pregnant women with micronutrient deficiency	19.3%
Presentation of pregnant women with anemia	40%
Babies born <2,500 g	10.2%
Babies born <48 cm	20.2%

Babies born weighing <2,500 g and length <48 cm are not necessarily referred to as neonatal stunting but as a dominant predictor of stunting at a later age (15). However, if the baby does not show significant changes in body weight and length until the age of 6 weeks, it can be categorized as neonatal stunting (29). Babies born weighing less than 2,500 g are 1.27–5.9 times more likely to experience growth retardation (15, 30). Children born less than 48 cm have a 15.0 times higher risk of stunting (30, 31).

Babies born to mothers who experience chronic malnutrition will have a higher risk of stunt growth than their peers (31, 32). Fetal growth is directly influenced by maternal nutrition through the availability of nutrients in the mother's body. Indirectly, maternal nutrition affects the fetal endocrine system and interferes with epigenetic development through gene regulatory activity. Currently, few studies analyze the effects of micronutrients on fetal growth, although maternal intake of certain micronutrients is known to affect fetal growth (10, 33). Vitamin D is a micronutrient that plays a role in neonates, especially in bone and jaw development (34). Pregnant women should have circulating vitamin D >40 ng/ml. Based on these needs, the recommendation for the Nutrition Adequacy Rate (RDA) from the Ministry of Health of the Republic of Indonesia in 2019, the recommended amount of vitamin D intake for pregnant women is around 15 μg or 600 IU per day. Inadequate vitamin D intake during pregnancy reduces fetal bone mineralization and the regulation of other mineral functions in the uterus, thereby affecting the health of the newborn, particularly fetal development and growth retardation (35, 36).

Another nutrient deficiency that affects the fetus and is also common in developing countries is vitamin A deficiency (37). Pregnant women with vitamin A deficiency are usually associated with iron deficiency anemia. Anemia pre-disposes pregnant women to infection, which then progresses to fetal IUGR. Babies will have lower hemoglobin levels than normal babies and are small for gestational age, with a high risk of infection (38, 39).

In summary, Table 2 shows micronutrient deficiency that affects fetal growth during pregnancy. These micronutrient deficiencies during pregnancy lead to several maternal pathologies prone to infections—intrauterine bacterial infections associated with most pre-term births. Viral infections, such as cytomegalovirus and rubella, are also associated with pre-term births, while hepatitis B virus leads to low birth weight (40).

**Table 2:** Micronutrient deficiency during pregnancy and their effects (28).

**Micronutrient**	**Effects on pregnant mother**	**Effects to fetal**
Vitamin A	Night blindness, increasing susceptibility to infection, high risk of pre-term labor, maternal anemia	IUGR, small for gestational age and birth weight, risk of fetal death due to infection
Vit B12	Anemia perniciosa	metabolism disturbance
Vit D	High risk of infection	IUGR, pre-mature birth, LBW, development defect of enamel, intrauterine bone growth disruption
Iron	Iron-deficiency anemia	The risk of pre-mature birth, LBW,
Zinc	High risk of infection due to immunity disturbance	Pre-mature birth, LBW, intrauterine immunity disturbance

## *In utero* dental development and association with the early-life course

Primary teeth begin to grow and develop at the end of the fifth week of pregnancy (41, 42). The formation of primary tooth enamel begins in the 11th−14th weeks of the fetus's life and is completed by the end of the third month. The initial stage includes matrix formation followed by uterine calcification (43). *In utero*, the central incisors begin to calcify at 15 weeks of gestation, and the lateral incisors begin to calcify 1 week later. For canines, this sequence continues weekly until calcification of the second primary molar begins with the proximal buccal spike at ~18–19 weeks. At birth, at 36 weeks *in utero*, the cusps and occlusal surfaces of the primary second molars are covered with a layer of calcified enamel, although a large amount of enamel plaque has formed before calcification is complete (44).

There are biochemically identifiable and visible stages of enamel formation; secretory stage: part of the mineral matrix is secreted. Enamel has a translucent appearance—transition phase: Replace degraded matrix with tissue fluid. Enamel has a translucent appearance. Maturity: The residual matrix is replaced by tissue fluid replaced by mineral uptake associated with crystal growth in width and thickness. Enamel has a white and porous (opaque) appearance (44). The first crystal is long, then wide and thick. During enamel maturation, inhibitory proteins necessary for sedimentation are completely removed. As the thickness of the matrix increases and the environment becomes suitable for crystallization, water and protein are absorbed. Kallikrein is important for removing proteins from tooth enamel during the maturation stage. Without kallikrein, the enamel proteins remain in the matrix, and the enamel prisms do not lock and coalesce, leading to enamel fracture and rapid loss of function. Amelogenin, which constitutes the majority (90%) of the organic matrix during the secretion phase, disappears during maturation while enamel persists. Enamel constitutes 10–15% of the developing enamel matrix, but it constitutes 50% of the total protein matrix in mature enamel (44).

In the early stages of tooth enamel formation, tooth enamel contains 20–30% protein. During mineralization, the protein content gradually drops to 7% at the beginning of the ripening stage. It is estimated that normal primary tooth enamel contains ~0.22% protein and 0.15% permanent tooth enamel, with a similar protein composition. The newly erupted tooth enamel surface is composed of water, proteins, and lipids, accounting for 12–14% of the volume (44).

Which defects appear during enamel formation depends on the ameloblast stage where the damage occurs. Enamel agenesis occurs due to cell damage during the secretory phase, whereas hypomineralization occurs during the secretory phase or the final maturation of enamel formation. Enamel is a stable structure, and defects in matrix secretion and maturation of primary teeth can serve as a permanent record of damage *in utero* or during the perinatal period. Any stressful events during pregnancy and childbirth can lead to metabolic changes in enamel formation, leading to clinical enamel defects. Babies born to mothers with compromised enamel formation due to complications during pregnancy are at high risk of developing dental caries in the early years (43).

Odontogenesis is a complex event in which cells undergo successive morphological and tissue differentiation such that changes in one cell population affect other cell populations. This complex process can lead to many possible disturbances in tooth development. Nutrient deficiencies such as vitamins A, B complex, D, E, and K in clinical and experimental studies lead to impaired tooth growth and development (45).

### Developmental defect of enamel

Enamel development defect (DDE) in primary teeth is a visible deviation from tooth enamel's normal appearance, which is translucent due to enamel organ damage during amelogenesis (43). This enamel development defect can be seen clinically as hypoplasia, a pre-disposing factor for early childhood caries (ECC) (46) and is associated with malnutrition (47); molar incisors hypo mineralization; amelogenesis imperfecta; and fluorosis (46). Primary teeth with imperfect calcification in the pit and fissure areas will facilitate the attachment and colonization of cariogenic bacteria so that ECC will develop more quickly on the tooth surface (48).

Amelogenesis of primary teeth begins at the 15th week of gestation and completes development around 12 months after birth (49). The risk of DDE is known to be related to social factors (50), nutritional factors (47), and infectious disease (51) that occurs during amelogenesis in the pre-and post-natal period. The highest prevalence is in infants born pre-maturely (<37 weeks gestation), and LBW (52). Concerning malnutrition, enamel hypoplasia occurs due to impaired ameloblast activity during the secretory phase of amelogenesis (53). Severity determines the extent of the defect partially formed enamel (53, 54). The exact mechanism of DDE and the etiologic factors are not known with certainty (54). Several longitudinal studies have shown that enamel hypoplasia is common in malnourished children living in economically disadvantaged communities (50).

### Enamel hypoplasia and early childhood caries

Pre-eruptive enamel hypoplasia and pre-carious dental lesions caused by plaque accumulation and cariogenic diet are sometimes difficult to distinguish (50). The two things often occur together, where the defect due to enamel hypoplasia occurs before or allows for the continuation of dental caries. Enamel hypoplasia occurs before tooth eruption so that when the primary tooth erupts into the oral cavity, the enamel structure is already damaged and susceptible to cariogenic bacteria attack (55). The irregularity of the smooth surface of the teeth that occurs as a result of enamel hypoplasia can trigger the colonization of cariogenic bacteria such as mutants streptococci (M.S.). Early colonization of M.S. in infants generally leads to an increased incidence of ECC. The relationship between M.S. and the incidence of ECC has been widely reported in several studies, including a recent study by Tanner et al. (56), and this situation is exacerbated by the baby's cariogenic diet (57).

Various diseases of the embryonic cells responsible for the formation of dentin and enamel can impair the formation of deciduous teeth intrauterine. This stigma stems primarily from covariates associated with low socioeconomic status and poverty, including malnutrition and other possible nutritional deficiencies, low birth weight and pre-term birth, prenatal infectious diseases, and many other risk factors affecting mothers and newborns. It affects various manifestations that lead to tooth decay; in most cases, the clinical manifestation is EHP. EHP is most commonly found on the upper front teeth of primary teeth but can extend to other teeth, including molars. Teeth with EHP are susceptible to early and heavy colonization by cariogenic bacteria, especially M.S. and Lactobacillus, which induce early caries in the enamel-deficient niche, leading to what we call HAS-ECC. However, initial colonization or increase of M.S. and other cariogenic bacteria is insufficient. EHP cannot develop into HAS-ECC without a caries-preventive diet containing fermentable carbohydrates. It is the duality of EHP and malnutrition that defines HAS-ECC. We further propose that the current definition of ECC includes not only one disease but multiple diseases, each with a distinct natural history, previous contributors, anatomical location, etiology, severity or invasion, chronic state, and chronic disease. Acute and spread among various racial/ethnic groups. The division between these groups was ambiguous due to similar etiologies, namely cariogenic bacteria and cariogenic diet. Some associate S-ECC with bottle-feeding and even longer breastfeeding habits, but some are more about correlation, not causation. We have defined HAS-ECC as a subgroup called S-ECC, but it is etiologically distinct from other possible causes of S-ECC (e.g., bottle caries), although we consider some cases associated with bottle feeding Has EHP potential, more specifically HAS-European Commission. The secretory or final maturation stage of tooth enamel formation (55).

## Linking fetal undernutrition that yields to neonatal stunting and early childhood caries

The start of fetal development is the thing that marks the beginning of life, as hypothesized by Barker in 1980. Chronic diseases that occur in adulthood are the implications of events during or immediately after birth. Based on this hypothesis, we also understand how the mother's nutritional status before and during pregnancy plays an important role in determining the weight and length of the baby born later and how the child's nutritional status later on. Several studies support this hypothesis by analyzing several chronic diseases suffered by adults related to children's health in early life (58). The association of health in early life with chronic disease also applies to dental and oral diseases, especially dental caries. Teeth are the strongest and strongest calcified body structures, but their formation is highly dependent on the influence of diet and metabolism of the fetus and immediately after birth. During pregnancy, the mother's health is as vital as the continuity of the formation of teeth (17).

Dental and oral health is closely related to general health and have been the center of attention of researchers for many years (59). Some systemic diseases have effects or manifestations in the oral cavity and are even detected early in these oral manifestations. Therefore, the nutritional status of pregnant women will be able to determine the dental health of their children. Various studies have shown a significant relationship between the history of the nutritional status of pregnant women and caries experienced by the child. Pregnant women who give birth in a state of malnutrition will have children with a 7.1 times risk of experiencing early childhood caries. Several studies have also shown that children with a history of low birth weight are more at risk of developing caries in their primary teeth early in life because of the abnormal calcification of the teeth during formation compared to children born with an average weight (60, 61).

The process of tooth mineralization begins at week 12, and this is a critical period because the enamel matrix will begin to form a network complex in the structure of primary teeth. In this process, macronutrients and micronutrients play a role so that if there is a deficiency, it will cause changes in tooth structure. Vitamin A, for example, plays an important role in maintaining the integrity and differentiation of epithelial cells. Vitamin A deficiency during this period will affect the activity of ameloblasts when the enamel is formed (62, 63). Vitamin A also affects growth by regulating growth hormone and thyrotropin beta genes so that its deficiency will cause a decrease in pituitary G.H. secretion and a decrease in fetal weight or survival. Research linking vitamin A deficiency with stunted growth, whether stunting or wasting, has yet to produce a definite answer. Several countries display different results, so the association between VAD and stunting should be population-specific rather than generalizing to the entire population (38, 39).

Vitamin D has a role that is closely related to growth, namely in the process of immune mechanisms through the metabolism of calcium and phosphorus, which are none other than minerals that are very important for teeth and bones (62, 63). The primary tooth matrix is attached during the pre-natal period, mediated by calcium and phosphate metabolism. Therefore, deficiency of some of these micronutrients will impair the integrity of the fetal teeth. Thus, pre-natal vitamin D intake can predict ECC occurrence in children. Pregnant women with high calcium intake were associated with a lower caries risk in children (*P* = 0.03) (63, 64). However, malnutrition that involves a lack of protein-energy also increases the risk of salivary gland dysfunction and enamel hypoplasia, which eventually causes caries in the primary teeth of children (65). The nutritional status of children in the first thousand days of life is related to dental and oral health (66, 67).

## Conclusion

The concept that links maternal malnutrition affecting fetal outcomes in neonatal stunting can affect the incidence of caries in early childhood. However, evidence of this direct relationship has not been obtained; comprehensive longitudinal studies are needed to support the conclusions in this mini-review. Since environmental factors can lead to enamel hypoplasia leading to persistent ECC, early detection of these enamel defects is critical to the understanding role of ECC etiology and to help implement effective strategies. Prevention is critical due to the nature of the enamel that can be strengthened and reinforced but cannot be replaced. The socialization of the first dental visit that both general practitioners and pediatricians can echo is important. This can ensure that information is conveyed on maintaining the infant's oral hygiene and health, in line with the child's general health in the future.

## Author contributions

AS made a substantial contribution to the concept or design of the article. RI, NS, LR, and NJ revised it critically for important intellectual content and approved the version to be published. All authors contributed to the article and approved the submitted version.

## Conflict of interest

The authors declare that the research was conducted in the absence of any commercial or financial relationships that could be construed as a potential conflict of interest.

## Publisher's note

All claims expressed in this article are solely those of the authors and do not necessarily represent those of their affiliated organizations, or those of the publisher, the editors and the reviewers. Any product that may be evaluated in this article, or claim that may be made by its manufacturer, is not guaranteed or endorsed by the publisher.
